# Highly Robust, Pressureless Silver Sinter-Bonding Technology Using PMMA Combustion for Power Semiconductor Applications

**DOI:** 10.3390/ma17215142

**Published:** 2024-10-22

**Authors:** Moses Gu, Hyunjin Nam, Sehoon Park, Minkyung Shin, Sung-Hoon Choa

**Affiliations:** 1 Intelligent Semiconductor Engineering Department, Seoul National University of Science and Technology, 232, Gongneung-ro, Nowon-gu, Seoul 01811, Republic of Korea; rnahtp123@seoultech.ac.kr; 2 Packaging Research Center, Korea Electronics Technology Institute, Seongnam-si 13509, Republic of Korea; 3 Electronic Convergence Materials and Devices Research Center, Korea Electronics Technology Institute, Seongnam-si 13509, Republic of Korea

**Keywords:** pressureless, sinter-bonding, PMMA, silver sintering

## Abstract

This study presents the development of a highly robust, pressureless, and void-free silver sinter-bonding technology for power semiconductor packaging. A bimodal silver paste containing silver nanoparticles and sub-micron particles was used, with polymethyl methacrylate (PMMA) as an additive to provide additional thermal energy during sintering. This enabled rapid sintering and the formation of a dense, void-free bonding joint. The effects of sintering temperature and PMMA content on shear strength and microstructure were systematically investigated. The results showed that the shear strength increased with rising sintering temperatures, achieving a maximum of 41 MPa at 300 °C, with minimal void formation due to enhanced particle necking facilitated by PMMA combustion. However, at 350 °C, the shear strength decreased to 35 MPa due to cracks and voids at the copper substrate–copper oxide interface caused by thermal expansion mismatch. The optimal PMMA content was found to be 5 wt.%, balancing sufficient thermal energy and void reduction. This pressureless sintering technology demonstrates significant potential for high-reliability applications in power semiconductor modules operating under high-temperature and high-stress conditions.

## 1. Introduction

The power modules used in eco-friendly vehicles, such as hybrid electric vehicles (HEVs), electric vehicles (EVs), and hydrogen fuel cell vehicles (FCEVs), operate at higher temperatures for longer durations compared to general electronic power modules. As a result, they require higher reliability under harsher environmental conditions [1]. High-performance power semiconductor devices and modules are essential for power conversion (DC↔AC), motor driving, switching, and control in these vehicles [2]. However, due to rapidly increasing technological demands, silicon-based power semiconductors are expected to soon reach their power density limits [3]. This increase in power density is predicted to raise the operating temperature from the current 150 °C to an environment of 200–300 °C [4,5]. Therefore, new materials and packaging technologies are required to meet the demands of power semiconductors operating in high voltage, current, and temperature environments. Wide-bandgap (WBG) semiconductors, such as silicon carbide (SiC) and gallium nitride (GaN), have garnered significant attention due to their ability to function at temperatures above 250 °C [4,6,7]. However, traditional die-attach materials like Pb-Sn and Sn-Ag-Cu solders lack reliability at these elevated temperatures due to their low melting points [6,8]. Furthermore, many solders contain lead (Pb), which poses significant environmental hazards [9].

To address these challenges, Ag paste-based sinter-bonding has emerged as an alternative die-attachment technology [10,11]. Nano-Ag particles exhibit excellent sintering properties at temperatures significantly lower than their melting point (200–300 °C). Additionally, sintered Ag joints, which have a melting point of 961 °C, maintain stable mechanical properties at temperatures above 300 °C [12,13,14]. Moreover, silver offers superior electrical and thermal conductivity [15]. It also benefits from a slower oxidation rate compared to other metals.

Due to these multiple advantages, several studies have actively investigated Ag paste for die-attachment. However, these studies typically utilize Ag nanoparticles to reduce the sintering temperature and time [16,17,18,19]. The use of Ag nanoparticles increases both material and manufacturing costs, while also introducing complex fabrication challenges, such as nanoparticle agglomeration during synthesis [16,20,21,22,23]. As a result, hybrid Ag pastes, composed of micro-Ag particles, Ag flakes, and chestnut-burr-like Ag particles, have been developed [24,25]. However, these formulations often require assisted pressure during sinter-bonding to achieve robust bonds, which involves additional manufacturing costs and the risk of chip damage or fracture due to the high bonding pressure [22,26,27].

To overcome these limitations, pressureless sinter-bonding technologies using Ag paste have been recently developed [16,28]. Although pressureless bonding offers advantages such as simplified automated manufacturing and reduced chip damage, several challenges remain. Specifically, pressureless bonding requires a relatively long bonding time (more than 30 min), and the resulting bond joints exhibited lower shear strength compared to pressure-assisted bonding [29,30,31]. For example, in previous studies, even when the bonding time exceeded 1 h at temperatures below 250 °C, the shear strength was lower than 30 MPa [32,33,34]. On the while, when the bonding time was reduced to less than 10 min at temperatures above 300 °C, the shear strength was measured to be below 14 MPa [35]. Furthermore, even when the bonding process was carried out at 350 °C for 30 min, the shear strength remained below 20 MPa [16]. While several efforts have been made to optimize pressureless Ag paste bonding processes [36,37], few studies have demonstrated the high-quality bonding required for semiconductor applications.

Ag pastes contain various organic components, including binders, solvents, dispersants, and additives. Organic residues remaining after pre-drying and sintering degrade the electrical and mechanical properties of the sintered Ag paste [38,39]. Additionally, outgassing from evaporating organic materials during sintering creates voids and pores in the bonding joint, reducing its strength and durability.

To address the challenges encountered in pressureless sinter-bonding processes, this study developed a bimodal silver paste for pressureless sintering, incorporating PMMA as an additive. The paste’s filler was composed of a mixture of Ag nanoparticles and sub-micro particles to enhance packing density. For the binder, polyvinylpyrrolidone (PVP) resin, known for its excellent reduction properties, and propylene carbonate, which thermally decomposes at temperatures below 200 °C, were used. PMMA, which exhibits combustibility at 280 °C, was employed as an additive to induce both internal and external sintering simultaneously at 300 °C, thereby reducing processing time efficiently. This approach enabled the formation of a robust bonding joint with reduced voids in a relatively short bonding time. Additionally, the effects of the PMMA additive on the shear strength and microstructure of the bonding joint were analyzed.

## 2. Materials and Methods

### 2.1. Preparation of the Ag Paste

For the Ag paste used for the pressureless sinter-bonding process, Ag nano particles were synthesized, and detailed fabrication processes were described in our previous study [40]. The silver nanoparticles used in this study were synthesized via the spray pyrolysis method. In this process, a solution of silver precursors was atomized into fine droplets and introduced into a high-temperature furnace (over 900 °C). The rapid thermal decomposition of the precursors in this high-temperature environment produced high-purity, highly crystalline silver particles. The spray pyrolysis method was chosen for its ability to yield silver particles with excellent electrical conductivity and minimal carbon contamination, making it suitable for low-temperature sintering applications.

**Figure 1:**
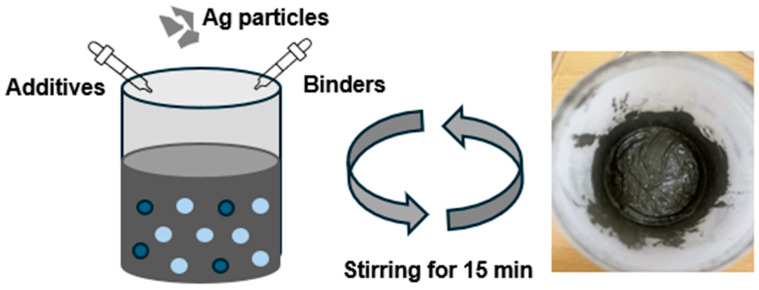
Schematic depicting the preparation of Ag Paste.

Using solely nano-silver particles lowers the melting point but reduces bonding strength and increases cost [14,24,41,42,43]. To compensate for those disadvantages, we manufactured Ag paste by combining submicron-sized silver particles and nanoparticles at a ratio of 3:7 wt.%. The average diameter of the nano Ag particles used was 70 nm (D50), and the average diameter of the submicron Ag particles was 700 nm (D50) (FTL-070N, FP Co., Ltd., Busan, Republic of Korea). Both types of the Ag particles were spherical shapes. The fabrication process of the Ag paste is depicted in Figure 1.

To fabricate the Ag paste, organic binders were added to the Ag particles to enhance printability and dispersibility during the stencil printing process. Table 1 shows the material composition and content ratios of the Ag paste in this study. Two types of binder materials were used: polyvinylpyrrolidone (PVP) (Sigma-Aldrich Inc., St. Louis, MO, USA) and propylene carbonate (Youngshin Co., Gimhae-si, Republic of Korea). PVP provides reduction properties and prevents oxidation during high-temperature processes under atmospheric conditions [44,45]. Propylene carbonate thermally decomposes at a relatively low temperature of 200 °C, evaporating during the Ag sintering process to minimize void formation. Additionally, propylene carbonate prevents the agglomeration of Ag particles and improves the dispersibility of the Ag paste. The binder mixing ratio was maintained at 1:1.

To further enhance the dispersibility of the Ag particles during the mixing process, 1 wt.% dispersant (DISPERBYK-111, BYK Co., Ltd., Wesel, Germany) was added. Ethylene glycol was selected to improve both storage stability and reducibility, as supported by previous studies [46,47]. Finally, 5 wt.% polymethyl methacrylate (PMMA) (LG MMA, Yeosu, Republic of Korea) was added to induce self-heating and internal sintering due to PMMA combustion during the pressureless sinter-bonding process. Mixing and dispersion of the Ag paste were carried out at 1300 rpm for 30 s using a high-speed paste mixer (PDM300, Daehwa Tech, Ulsan, Republic of Korea).

To investigate the effects of the PMMA additive on the microstructure of the Ag paste and the shear strength of the bonded chip, different contents of PMMA were added to the Ag paste, 0 wt.%, 2 wt.%, 5 wt.%, and 7 wt.%.

### 2.2. Sinter-Bonding Process

**Figure 2:**
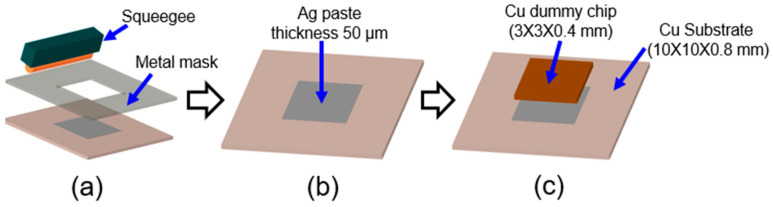
Schematic drawing of Ag pressureless sintering process: (**a**) stencil-printing of Ag paste, (**b**) implementing the pre-drying process, (**c**) chip mounting on applied Ag paste.

**Figure 3:**
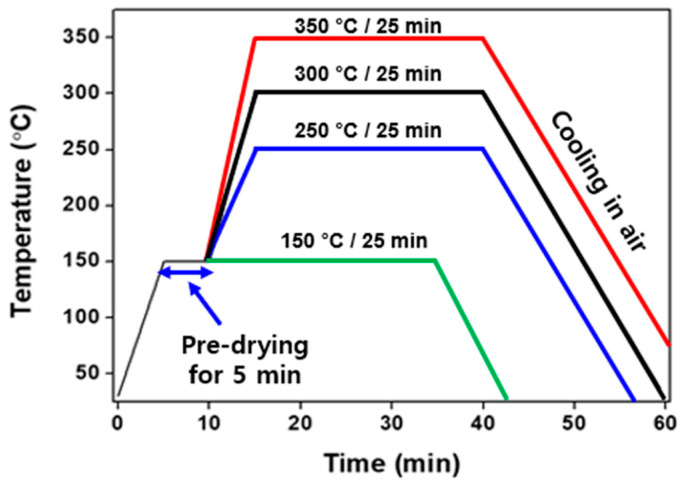
Bonding temperature profile.

Figure 2 shows a schematic drawing of the sinter-bonding process. To perform chip bonding using Ag paste, a bare Cu substrate with 99.99% purity and dimensions of 10 × 10 × 0.8 mm (length, width, and thickness) and a Cu dummy chip of 3 × 3 × 0.4 mm were used. Prior to bonding, the bare Cu substrate and Cu chip were polished with #2000 abrasive paper and then cleaned in an ultrasonic alcohol bath for 5 min. As shown in Figure 2a, the produced bimodal silver paste was uniformly stencil-printed to a thickness of 50 μm using a squeegee with a metal mask on the Cu substrate. Then, the Cu dummy chip was mounted on the stencil-printed Ag paste as illustrated in Figure 2b. Afterwards, a pre-drying process was implemented to evaporate the solvent in the paste using a hot plate at 150 °C for 5 min in the air without assisted pressure. Lastly, as shown in Figure 2c, the Cu chip mounted on the substrate underwent a pressureless sinter-bonding process in a reflow oven. The temperature profile of the bonding process was established and is shown in Figure 3. The bonding was conducted at different temperatures of 150 °C, 250° C, 300 °C, and 350 °C for 25 min, respectively.

### 2.3. Characterization and Test Methods

The sintering temperature range (150 °C to 350 °C) was selected based on the thermal properties of the Ag paste and the decomposition behavior of its organic additives. Thermogravimetric analysis (TGA) and differential thermal analysis (DTA) revealed that significant thermal events, such as the evaporation of propylene carbonate and the combustion of PMMA, occur within these temperature ranges. Temperatures below 150 °C were insufficient to initiate sintering and neck formation between silver particles, while temperatures above 350 °C risked excessive oxidation of the copper substrate, potentially degrading the joint’s mechanical properties due to thermal expansion mismatch.

Thermogravimetric (TG) and differential thermal analysis (DTA) were conducted using a DTG-60H thermal gravimetric analyzer (Shimadzu Co., San Jose, CA, USA), with the temperature increased at a rate of 10 °C per minute from room temperature to 500 °C under atmospheric conditions. These tests were performed to better understand the sintering behavior of the Ag paste and determine the optimal bonding temperature profile based on the thermal decomposition characteristics of the binders and additives.

The surface morphology and cross-sectional images of the sintered Ag paste and bonding joints were analyzed using a scanning electron microscope (SEM) with a focused ion beam (FIB) (XII Vision 200TB, SEIKO Corp., Tokyo, Japan). The bonding strength of the Cu joint after pressureless sintering was evaluated using a shear tester (Dage 4000, Nordson Co., Westlake, OH, USA), with the shear test conducted at a speed of 100 μm/s. Additionally, the Cu substrate with the bonded dummy chip was epoxy-molded and polished using an IM4000plus CTC (Hitachi Ltd., Tokyo, Japan) to examine the cross-section of the bonding joint. Both the cross-section and the fractured surface of the bonded chip after shear testing were further analyzed using a scanning electron microscope (SU-8010, Hitachi Ltd., Japan). Elemental distribution on the fractured surfaces was examined using an energy-dispersive spectrometer (EDS).

## 3. Results and Discussion

### 3.1. Sintering and Bonding Process

**Figure 4:**
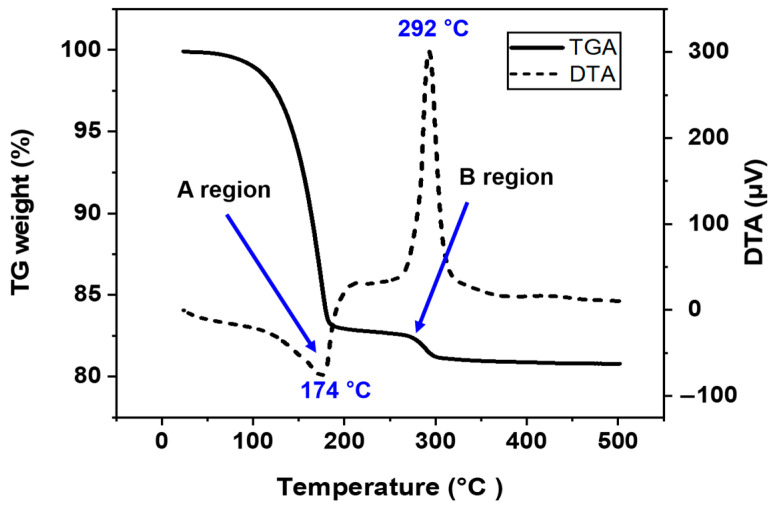
TG-DTA curves of Ag paste in atmospheric conditions.

Figure 4 shows the TG-DTA curve of the bimodal Ag paste measured as a function of temperature. This measurement was conducted to obtain a better understanding of and optimize sintering temperatures during the pressureless bonding process. The solid line represents the relative weight with TG analysis, and the dotted line represents the results of the DTA analysis. When the temperature reaches 174 °C, weight loss occurs along with endothermic reaction (A region in Figure 4), indicating that most organic substances in the paste have been evaporated, except the Ag particles and PMMA, which have higher melting points. In particular, the propylene carbonate binder evaporates together with highly volatile ethylene glycol by forming an azeotrope at 174 °C. As the temperature increases to 292 °C, the Ag paste experiences additional weight loss (B region), showing a sharp exothermic peak in the DTA curve, which is attributed to the burning of PMMA since the flash point temperature of PMMA is 280 °C. There is no further weight loss and reaction above 300 °C, so it is thought that all organic materials in the Ag paste have been completely evaporated.

**Figure 5:**
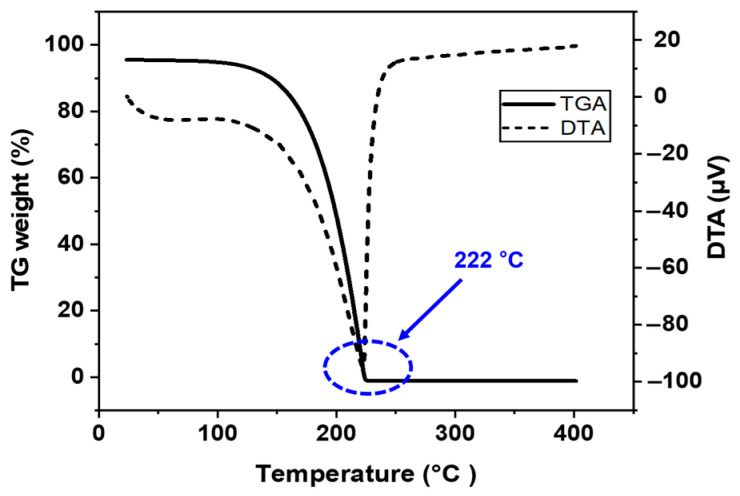
TG-DTA curves of propylene carbonate in atmospheric conditions.

Figure 5 shows the TG-DTA analysis results of the propylene carbonate binder material in the Ag paste. When the temperature reaches 222 °C, there is a weight loss of 99.777% simultaneously with an endothermic reaction, indicating that most of the propylene carbonate evaporates at this temperature. Minimizing the residual components in the paste is very important to enhance the bonding strength of the bonding joint as well as increase electrical conductivity, by increasing intermetallic necking between the Ag particles [39].

**Figure 6:**
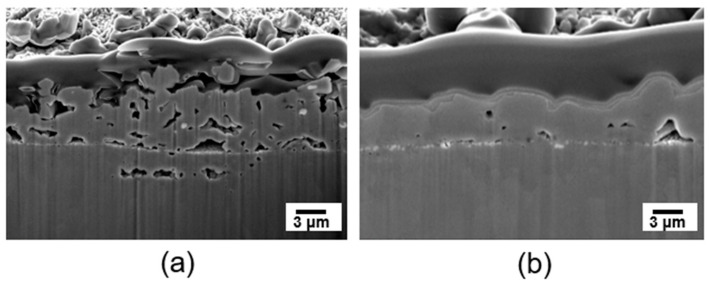
Cross-sectional SEM images: (**a**) sintered at 300 °C without pre-drying and (**b**) sintered at 300 °C pre-drying at 150 °C for 5 min.

The pre-drying process before sintering the Ag paste plays an important role. Figure 6 exhibits images of the cross-sectional microstructure of the bonding joint. Figure 6a shows an image of the bonding joint when the sintering process was performed at 300 °C without pre-drying. Several large voids, including the largest pores with 0.9 μm in length and 0.3 μm in height, can be observed in the bonding joint. In the sample sintered at 300 °C without pre-drying, large voids are visible, likely caused by the rapid outgassing of evaporating organic components during sintering [48]. Figure 6b is a cross-sectional image of the bonding joint sintered at 300 °C after the pre-drying process was conducted at 150 °C. Only a few voids can be observed. During the pre-drying process, the organic materials in the Ag paste evaporated and escaped outside. Afterwards, necking between Ag particles occurred, resulting in a denser structure. For the sample sintered at 300 °C, the electrical resistivity of the sintered Ag was measured using a four-point probe system. The sintered Ag paste showed a relatively low resistance value of 1.40 × 10^−5^ Ω·cm, comparable to that of the Ag pastes reported in several previous studies [35,49,50].

### 3.2. SEM Analysis of Sintered Ag Paste

**Figure 7:**
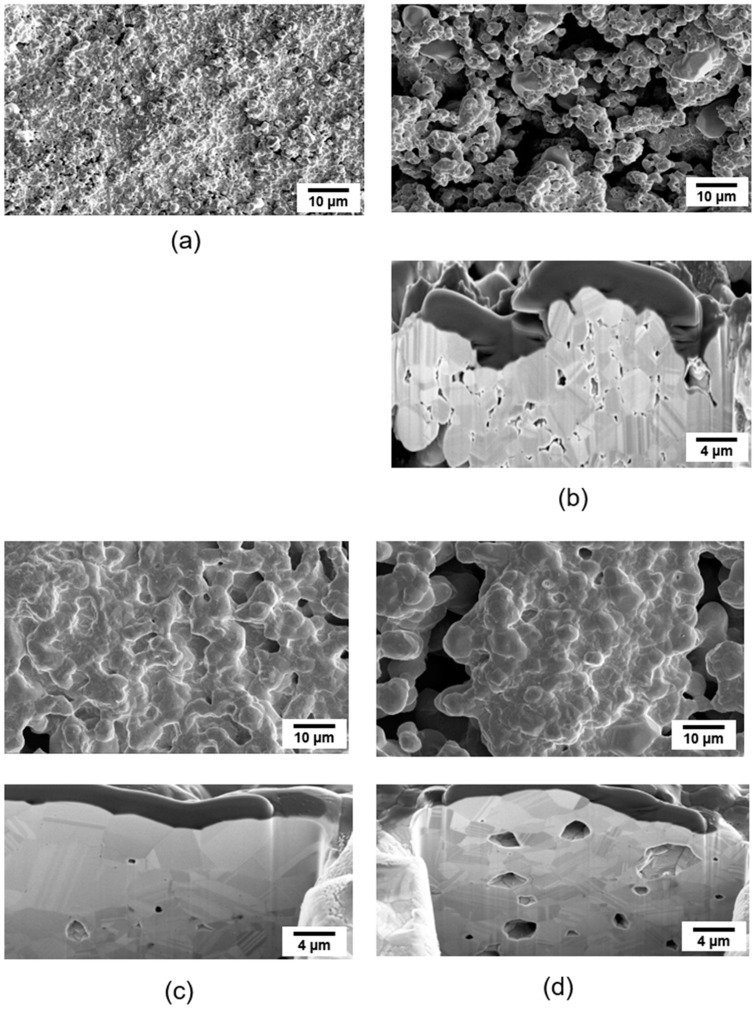
SEM images of surface of sintered Ag paste sintered at (**a**) 150 °C and SEM images of surface and cross-section of sintered Ag paste sintered at (**b**) 250 °C, (**c**) 300 °C, and (**d**) 350 °C.

Figure 7 exhibits SEM images of the Ag pastes. Figure 7a shows the surface morphology of the Ag paste after pre-drying at 150 °C for 5 min without sintering. The SEM image shows the uniform distribution of both nano and sub-micro Ag particles after pre-drying at 150 °C for 5 min, but no particle necking was observed at this stage. Figure 7b–d shows SEM images of the Ag pastes which were pre-dried at 150 °C and subsequently sintered at different temperatures of 250 °C, 300 °C, 350 °C, respectively. The top images show the surface morphology, and the bottom images show the cross-sectional images of the bonding joint of the Ag paste. As shown in Figure 7a, when only the pre-drying process was conducted, the nano Ag particles and sub-micro Ag particles were uniformly distributed, however, necking between particles did not proceed.

Figure 7b shows the images of the Ag paste sintered at 250 °C. At 250 °C, nano Ag particles began sintering and forming necks with sub-micro Ag particles, but rapid evaporation of organics inhibited further necking and caused porosity. The rapid evaporation of the organics prevents the formation of necking and reduces the bonding strength of the bonded joint. As shown in Figure 7c, when the Ag paste was sintered at 300 °C, the Ag paste had more coarse necking and a denser microstructure. Also, the number of voids in the bonding joint was significantly reduced. As can be seen from the DTG results in Figure 4, when the sintering temperature reaches 292 °C, the PMMA in the Ag paste begins burning. At 300 °C, the exothermic reaction due to the burning of PMMA supplies additional thermal energy, facilitating sintering and enhancing the material’s packing density.

Figure 7d displays the SEM images of Ag pastes sintered at 350 °C. The surface morphology and microstructure of the Ag paste were similar to those sintered at 300 °C. However, some large voids were observed. When the temperature increased sharply to 350 °C for 25 min, necking between the Ag particles was initiated before the residual organic substances in the Ag paste evaporated and escaped, resulting in the formation of voids in the Ag paste.

### 3.3. Shear Test Results

**Figure 8:**
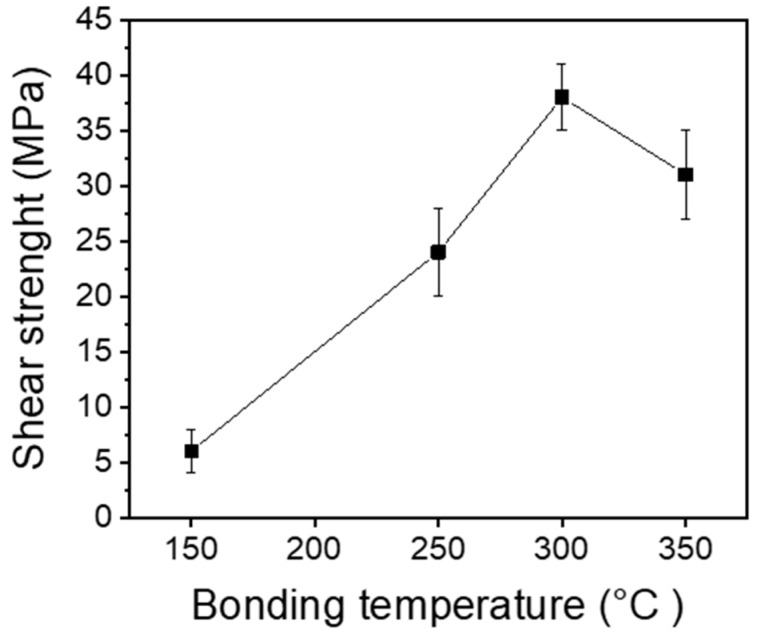
Shear strength results of each bonding temperature.

Figure 8 exhibits the shear test results of Ag pastes bonded at different sinter-bonding temperatures of 150 °C, 250 °C, 300 °C, and 350 °C. The shear strength of the Ag paste increased linearly with increasing sintering temperature. The samples bonded at 150 °C showed an average shear strength of less than 10 MPa, which was attributed to the incomplete sintering and poor necking of Ag particles at this temperature. The samples bonded at 250 °C showed a maximum shear strength of 27 MPa, which was higher than that of the samples sintered at 150 °C. The shear strength value of 27 MPa is also like that of Pb-5Sn solders used in semiconductor power modules [51].

The Ag pastes sintered at 250 °C had several small voids in the bonding joint, as shown in Figure 7b, which are detrimental to the robustness and reliability of the bonding joint. However, the increase in shear strength at 250 °C is since the propylene carbonate binder in the Ag paste was thermally decomposed at a relatively low temperature and evaporated at about 225 °C. As a result, necking between Ag particles in the Ag paste can actively occur at 250 °C, resulting in a relatively dense and uniform sintered bonding joint, with a comparably high bonding strength.

The Ag paste bonded at 300 °C exhibited the highest shear strength of 41 MPa. As the sintering temperature increased to 300 °C, the diffusion of Ag particles was facilitated, and the contact area between particles increased, leading to the formation of a denser microstructure joint at the bonding interface, thereby improving mechanical strength. In particular, the sharp increase in shear strength was attributed to a denser microstructure and necking, with less voids, as shown in Figure 7c, due to the self-heating and additional thermal energy during burning of PMMA at 292 °C. In summary, we achieved robust pressureless Ag sintered bonding with a comparable short bonding time of 30 min.

Meanwhile, when the sintering temperature increased to 350 °C, the shear strength was reduced to 35 MPa. Bimodal Ag pastes tend to exhibit higher bonding strength at elevated sintering temperatures due to improved particle diffusion and necking between nano and sub-micron Ag particles [33,52]. As the temperature increases, the diffusion between nano Ag particles and sub-micron Ag particles is promoted, thereby increasing necking and forming a dense Ag porous structure, leading to an increase in bonding strength. However, in this study, the shear strength decreased at 350 °C. To investigate the cause of the decrease in shear strength at 350 °C, the bonding joints of the samples bonded at 300 °C and 350 °C were investigated by SEM and EDS mapping analysis, respectively.

### 3.4. SEM Analysis of the Cross-Sectional Bonding Area

**Figure 9:**
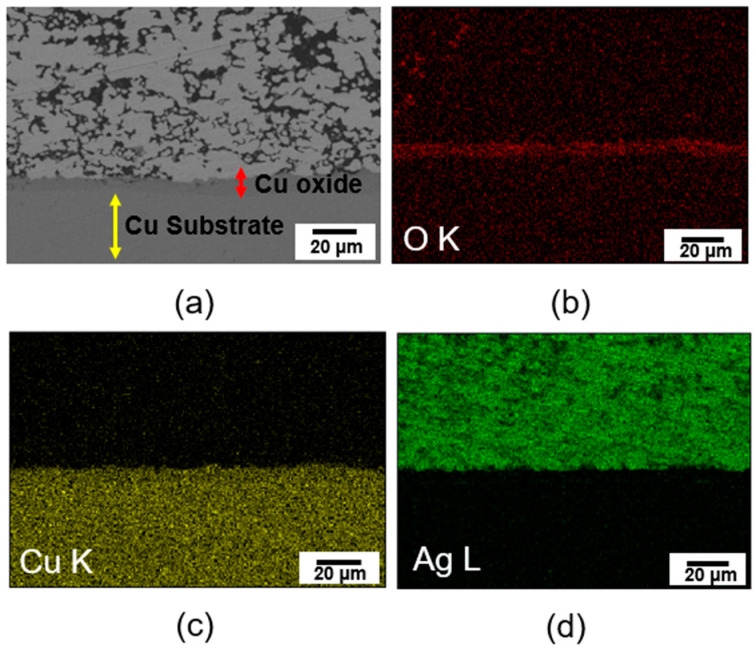
Analysis of cross-sectional bonding area sintered at 300 °C: (**a**) SEM image of cross-sectional bonding area, (**b**–**d**) EDS elements’ mapping results of cross-sectional bonding area.

Figure 9a shows a cross-sectional SEM image of the bonding joint bonded at 300 °C. An oxide layer is visible between the Ag paste and the Cu substrate. It was observed that the Ag paste, Cu oxide, and Cu substrate were tightly bonded, without voids and cracks between the interface structure. The existence of the oxide layer was confirmed by EDS mapping analysis. Figure 9b–d shows the EDS mapping images. As shown in Figure 9b, A thin Cu oxide layer was uniformly formed at the interface, tightly bonding the Ag paste to the Cu substrate without voids or cracks. oxygen (O) element was detected in the bonding joint, and the distribution of the O element matched the distribution and location of the Cu element, as shown in Figure 9c. SEM and EDS mapping results indicated that a thin copper oxide layer was uniformly formed at the interface between the sintered Ag and Cu substrate without voids.

**Figure 10:**
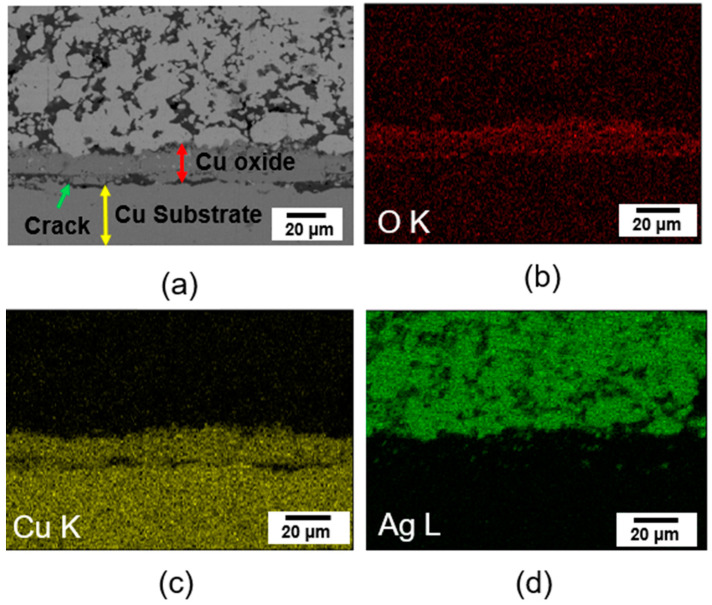
Analysis of cross-sectional bonding area sintered at 350 °C: (**a**) SEM image of cross-sectional bonding area, (**b**–**d**) EDS elements’ mapping results of cross-sectional bonding area.

Figure 10a shows a cross-sectional SEM image and Figure 10b–d shows the EDS mapping images of the bonding joint bonded at 350 °C. Several pores and cracks can be observed, particularly at the interface between the Cu oxide and Cu substrate. As shown in Figure 10b,c, a thick O element distribution was detected, indicating that the thick Cu oxide layer grew at the interface between the Cu substrate and sintered Ag due to the high bonding temperature of 350 °C.

At 350 °C, oxygen diffused through the porous structure of the sintered Ag, leading to the oxidation of the Cu substrate and the formation of a thick oxide layer [34]. A relatively thin oxide layer was formed during the bonding process at temperatures below 300 °C since ethylene glycol and the PVP additives act as Cu reducing agents. As shown in Figure 10a, cracks and voids occurred at the interface structure between the Cu substrate and Cu oxide layer. It is thought that the cracks were caused by the mismatch in the thermal expansion coefficient (CTE) of the Cu (16.5 × 10^−6^ C^−1^) substrate and Cu_2_O (1.59 × 10^−6^ C^−1^) oxide layer [53,54]. A CTE mismatch will induce interface delamination of the bonding joint, leading to a reduction in the shear strength of the bonding joint.

### 3.5. Analysis of the Fractured Surface of the Bonding Joint

**Figure 11:**
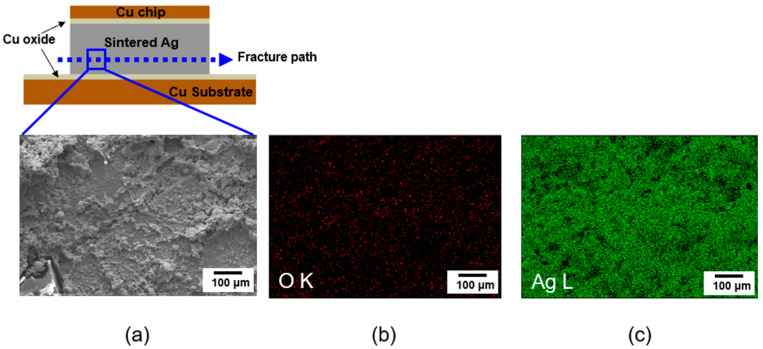
(**a**) Schematic drawing of the fracture path and SEM image, (**b**,**c**) EDS mapping results of the fracture surface of joints sintered at 300 °C.

To investigate the change in shear strength with different bonding temperatures, the fractured surfaces of the samples were analyzed after the shear tests. Figure 11 exhibits the SEM image and EDS mapping images of the fractured surface for the sample bonded at 300 °C. Figure 11a exhibits a schematic drawing illustrating the fracture path and SEM surface image of the fractured surface, and Figure 11b,c shows the results of the EDS mapping. The fracture path represents the weak point in the bonding joint. Ag element was detected in the fractured surface; however, the Cu element was not detected, indicating that the fracture passed through the inside of the sintered Ag paste during the shear test. These results also demonstrate that the bonding interface between the Ag sintered paste and Cu substrate is very robust with very high adhesion strength.

**Figure 12:**
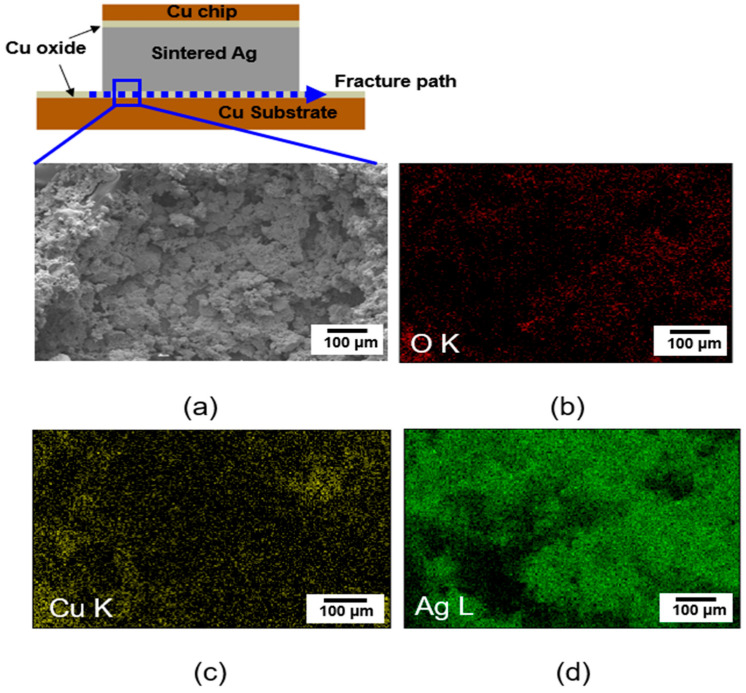
(**a**) Schematic drawing of the fracture path and SEM image, (**b**–**d**) EDS mapping results of the fracture surface of joints sintered at 350 °C.

Figure 12a shows a schematic drawing of the fracture path, and a SEM surface image of the sample bonded at 350 °C, and Figure 12b–d shows the EDS mapping results of the fractured surface. As shown in Figure 12c, the Cu element was detected at the fractured surface, indicating that the fracture occurred at the interface between the sintered Ag paste and the Cu substrate.

It is noted that the fracture path of the sample bonded at 350 °C was different from that of the sample bonded at 300 °C. As shown in Figure 10a, for the sample bonded at 350 °C, cracks and voids were observed at the interface between the Cu substrate and Cu oxide layer, caused by the CTE mismatch between the Cu substrate and Cu oxide layer. Accordingly, the interface between the Cu substrate and Cu oxide layer is a region more vulnerable to shear stress. The sintered bonding joint consists of a sandwich structure (Cu chip-sintered Ag paste/Cu substrate). It is expected that the interface between the Cu chip and sintered Ag paste could be another region vulnerable to shear stress.

However, the reason why the fracture occurred mainly at the interface between the sintered Ag and Cu substrate may be related to the size effect, since the Cu substrate (10 × 10 × 0.8 mm) is larger than the Cu chip (3 × 3 × 0.4 mm). Due to the large area of the Cu substrate, there can be greater chances of cracks and voids at the interface between the sintered Ag and the Cu substrate, so that the bonding strength at this region will be weaker than the interface between the Cu chip and the sintered Ag. For this reason, the fracture occurred at the interface between the Ag sintered paste and Cu substrate.

### 3.6. Effects of PMMA Contents

**Figure 13:**
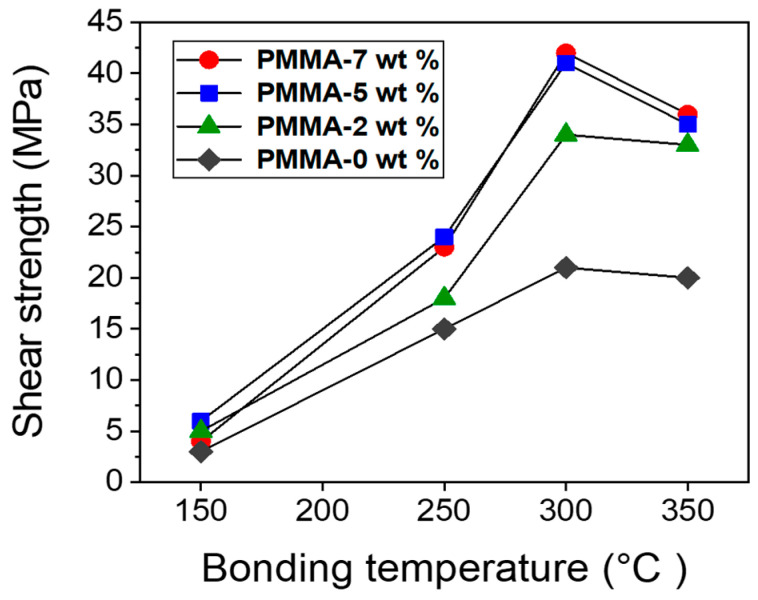
Result of shear strength test based on PMMA concentration.

In this study, Ag pastes were fabricated with different contents of PMMA to explore the effects of the additional thermal energy contributed by the burning PMMA during the sintering process. Ag pastes were fabricated with different contents of PMMA, of 0 wt.%, 2 wt.%, 5 wt.%, and 7 wt.% in the Ag paste. After fabricating the Ag pastes with different contents of PMMA, and then pressureless bonding process was conducted at different bonding temperatures of 150 °C, 250 °C, 300 °C, and 350 °C with a bonding time of 30 min. Figure 13 exhibits the results of the shear tests of each Ag paste bonded at different temperatures.

When the Ag pastes were bonded at a temperature of 150 °C, the maximum shear strength of the Ag pastes was less than 6 MPa for all samples, regardless of the content of PMMA, indicating the bonding temperature was too low to promote dense necking between Ag particles. It was also observed that that the Ag paste fabricated without PMMA (0 wt.%) showed the lowest shear strength among the samples fabricated with different PMMA contents. As the PMMA contents increased, the shear strength generally increased.

At the same time, the shear strength of the Ag paste with 5 wt.% PMMA was almost the same as that of Ag paste with 7 wt.% PMMA. When the bonding temperature rose to 250 °C, the shear strength of all samples increased significantly, and the shear strength of the Ag pastes with 5 wt.% and 7 wt.% PMMA content were 33% higher than that of the Ag paste with 2 wt.% PMMA. As the bonding temperature increased to 300 °C, the shear strength of the sample increased sharply. As the content of PMMA increased at a bonding temperature of 300 °C, the shear strength of the sample increased significantly. The sample without PMMA had a shear strength of 21 MPa, while the sample with 7 wt.% PMMA showed the highest shear strength of 42 MPa, which is 95% higher shear strength than the sample without PMMA. This higher shear strength was attributed to the formation of stable and robust necking between particles due to the additional thermal energy generated by burning PMMA during bonding. At the bonding temperature of 350 °C, the shear strength of the bonded chip tended to slightly decrease, regardless of PMMA content. As previously noted, this phenomenon was caused by the generation of cracks and voids at the interface due to the CTE mismatch between the Cu substrate and Cu oxide, resulting in a decrease in shear strength, as shown in Figure 10a.

**Figure 14:**
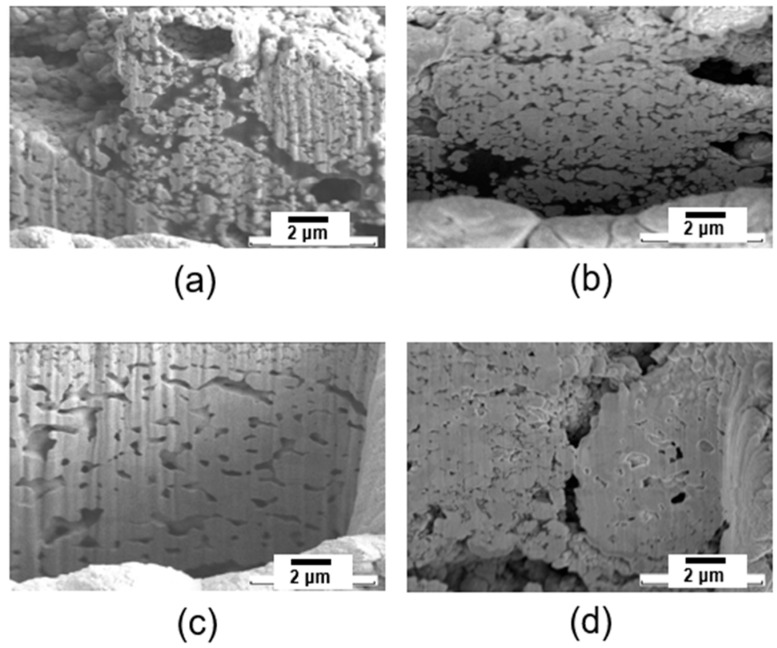
SEM images of the partial view, in cross-section, of sintered Ag paste at different PMMA concentrations. (**a**) Ag paste PMMA-0, (**b**) Ag paste PMMA-2, (**c**) Ag paste PMMA-5, and (**d**) Ag paste PMMA-7.

Figure 14 shows cross-sectional FIB images of the bonding joint for the samples fabricated with different PMMA contents which were bonded at 300°. For the Ag paste sample fabricated without PMMA, in Figure 14a, necking between Ag particles only partially formed, and nano Ag particles were located between the sub-micron Ag particles. The necking was interrupted by the remaining organic components, and some Ag particles retained their initial shape and morphologies. Some voids and pores are also observed.

Figure 14b exhibits the FIB image of the bonding joint of the Ag paste with 2 wt.% PMMA. It shows a relatively dense necking formation, and integration of the nanoparticles and microparticles, due to the external thermal energy generated by PMMA burning. The residual organic components were also significantly reduced. However, some isolated Ag particles without bridging were observed between the bulk particles forming the sintered necking. Figure 14c displays an image of the bonding joint for the Ag paste with 5 wt.% PMMA. The Ag nanoparticles and microparticles have merged into the porous structure, forming a considerably dense and uniform structure while all organic components were removed, due to the rapid decomposition of organic components during the burning of PMMA, resulting in a high shear strength of 41 MPa as shown in Figure 12. Figure 14d exhibits the image of the Ag paste with 7 wt.% PMMA. A dense Ag porous structure with no residual organic component was also observed. However, due to the high PMMA content, large voids were observed locally due to the excessive burning of PMMA during sintering. The shear strength of the Ag paste with 7 wt.% PMMA was almost the same as that of Ag paste with 5 wt.% PMMA. Therefore, 5 wt.% PMMA would be the optimal amount to add into the paste in terms of the material cost.

## 4. Conclusions

In this study, a highly robust, pressureless, and void-free silver sinter-bonding technology was developed using a bimodal silver paste containing silver nanoparticles and sub-micron particles. We also utilized the organic components to obtain a robust sinter-bonding. Propylene carbonate binder which thermally decomposes at a relatively low temperature of 200 °C, evaporating during the Ag sintering process was used to minimize void formation. The incorporation of PMMA as an additive provided additional thermal energy during the sintering process, enabling rapid sintering and the formation of a dense, void-free bonding joint. The shear strength increased with the sintering temperature, reaching a maximum of 41 MPa at 300 °C, attributed to enhanced particle necking and reduced void formation facilitated by the exothermic reaction of PMMA. However, at 350 °C, the shear strength decreased to 35 MPa due to the formation of cracks and voids at the interface between the copper substrate and copper oxide layer, caused by the mismatch in their coefficients of thermal expansion (CTE). The optimal PMMA content was determined to be 5 wt.%, as it provided a balance between sufficient thermal energy and minimal void formation.

## Figures and Tables

**Table 1:** Composition of Ag paste.

	Filler	Resin	Additive
wt.%	87	6	7
Material	Ag particles	PVP	Dispersant
		Propylene carbonate	PMMA
			Ethylene Glycol

## Data Availability

The original contributions presented in the study are included in the article, further inquiries can be directed to the corresponding author.
